# Molecular Detection of Respiratory Tract Viruses in Chickens at the Point of Need by Loop-Mediated Isothermal Amplification (LAMP)

**DOI:** 10.3390/v16081248

**Published:** 2024-08-03

**Authors:** Mohamed El-Tholoth, Haim H. Bau

**Affiliations:** 1 Department of Virology, Faculty of Veterinary Medicine, Mansoura University, Mansoura 35516, Egypt; 2 Veterinary Sciences Program, Health Sciences Division, Al Ain Men’s Campus, Higher Colleges of Technology, Al Ain 17155, United Arab Emirates; 3 Department of Mechanical Engineering and Applied Mechanics, University of Pennsylvania, Philadelphia, PA 19104, USA; bau@seas.upenn.edu

**Keywords:** chickens, LAMP, molecular diagnosis, point of need, respiratory diseases, rapid detection

## Abstract

Accurate and timely molecular diagnosis of respiratory diseases in chickens is essential for implementing effective control measures, preventing the spread of diseases within poultry flocks, minimizing economic loss, and guarding food security. Traditional molecular diagnostic methods like polymerase chain reaction (PCR) require expensive equipment and trained personnel, limiting their use to centralized labs with a significant delay between sample collection and results. Loop-mediated isothermal amplification (LAMP) of nucleic acids offers an attractive alternative for detecting respiratory viruses in broiler chickens with sensitivity comparable to that of PCR. LAMP’s main advantages over PCR are its constant incubation temperature (∼65 °C), high amplification efficiency, and contaminant tolerance, which reduce equipment complexity, cost, and power consumption and enable instrument-free tests. This review highlights effective LAMP methods and variants that have been developed for detecting respiratory viruses in chickens at the point of need.

## 1. Introduction

Respiratory viruses of chickens like infectious bronchitis virus (IBV), Newcastle disease virus (NDV), infectious laryngotracheitis virus (ILTV), avian influenza virus (AIV), and avian metapneumovirus (aMPV) are major causes of morbidity, mortality, and loss of productivity in global poultry farming with severe economic consequences [1]. The expenses related to controlling these diseases, along with the high mortality rates, can be overwhelming. Importantly, the current avian flu outbreak in the United States has caused losses ranging from USD 2.5 to USD 3 billion by the end of 2023, and these expenses are anticipated to rise with subsequent waves of the disease [2]. Moreover, decreased egg production, weight loss, compromised feed conversion rates, and immunosuppression compound the economic challenges for poultry producers and adversely impact food security [1,2,3,4]. Some of these viruses, such as NDV and AIV, also pose zoonotic risks, raising concerns not only for poultry but also for public and farm animals’ health. These viruses can infect chickens of all ages and may interact with bacterial pathogens, exasperating the situation [5,6]. With global warming and climate change, the enormity of this problem is expected to increase.

Rapid identification of these respiratory viruses would allow for effective control measures and reduced production losses. Currently, diagnosing viral respiratory diseases in the field relies on observing clinical signs and examining post-mortem lesions, which are not definitive and necessitate confirmation through laboratory tests. Confirming the presence of the virus typically occurs in centralized labs, often remote from the farms, through virus isolation (VI), enzyme-linked immunosorbent assay (ELISA), hemagglutination inhibition (HI), immunofluorescence, virus neutralization (VN) tests, conventional PCR, and quantitative PCR (qPCR) [7,8,9,10,11,12,13,14,15,16]. However, these diagnostic techniques are time-consuming and require sophisticated equipment and skilled personnel—all of which are absent from most farms. Quick, specific, and straightforward detection is needed in basic veterinary diagnostic labs, especially in resource-limited settings.

The LAMP method for amplifying nucleic acids could quickly identify viruses in the chicken respiratory system, offering sensitivity comparable to PCR [17,18,19,20,21]. Since LAMP employs a DNA polymerase with strand displacement activity, it does not require temperature cycling. It can be carried out at a constant incubation temperature, simplifying instrumentation, reducing energy consumption, and being amenable to electricity-free incubation. LAMP produces an order of magnitude more amplicons than PCR, enabling the simple monitoring of the amplification process with and without instruments [22,23,24,25,26,27].

The LAMP reaction can be adapted in microfluidic formats, like small plastic cartridges containing microscale fluid circuits for both sample processing and analysis [28,29]. These microfluidic chips offer advantages such as small reagent volumes, reduced cost, automated operation, portability, sample containment, and user-friendliness, making them accessible to non-experts. Single-use (disposable) chips can integrate multiple unit operations, such as lysis and nucleic acid extraction, for nucleic acid amplification tests [30,31,32].

Diverse avian viruses causing respiratory infections present similar clinical symptoms but may require different control strategies. Therefore, there is a need for multiplexed assays to co-detect co-endemic pathogens simultaneously. Multiplexing can be achieved in a few ways, such as single-pot multiplexing, multiple LAMP reaction chambers, and two-stage amplification (Penn-RAMP) [33,34,35]. A single pot may include primers for different targets and sequence-specific fluorescent probes that emit at different wavelengths (colors). This single-pot approach is limited in the number of targets that can be co-amplified and requires a sophisticated reader for multi-color detection.

Alternatively, one can aliquot a single sample into multiple LAMP reaction chambers, each specialized (with pre-stored primers) to amplify a single or a group of targets. This approach enables the use of a single-color dye, simplifying the reader and even enabling reader-free colorimetric detection. The need, however, to aliquot a sample into multiple reaction chambers necessitates dilution of the number of templates available for amplification in any reactor, adversely affecting sensitivity. Penn-RAMP [34] cures this deficiency. Penn-RAMP comprises two nested amplification steps. The first stage of Penn-RAMP is (reverse-transcriptase) recombinase polymerase amplification (RT-RPA) at 38 °C–42 °C. The second stage of Penn-RAMP is LAMP at ~65 °C. The first stage includes primers for all targets of interest (up to 16 have been demonstrated) and is carried out for a relatively short time, enough to increase the number of targets for the second stage greatly but not to produce any detectable signal. The products of the second stage are then (self) aliquoted into multiple LAMP reaction chambers, each specialized to amplify a single or a group of targets. Serendipitously, Penn-RAMP offers significantly improved sensitivity over stand-alone LAMP, qPCR, and RPA. A single-plex, two-stage Penn-RAMP has been demonstrated for avian virus detection in a closed tube [36].

**Figure 1:**
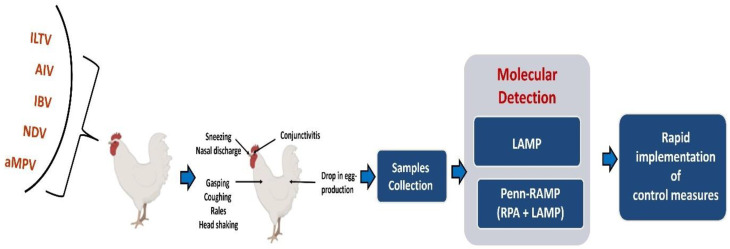
Workflow for molecular diagnosis of respiratory tract viruses in chickens by LAMP and Penn-RAMP.

LAMP and Penn-RAMP hold promise for clinical molecular diagnosis and virus surveillance (Figure 1) in resource-poor settings, including respiratory diseases in chickens, without requiring advanced equipment or highly trained personnel. This review showcases LAMP and Penn-RAMP techniques that have been developed thus far for detecting respiratory viruses in chickens. Furthermore, these devices can be linked to smartphone and mobile electronics to facilitate real-time spatiotemporal surveillance and predictions of disease spread trajectories—something that increases in importance with climate changes and the spread of vectors to new regions.

## 2. Standalone LAMP Method

Notomi et al. developed the LAMP method, which was subsequently patented by Eiken Chemical Co., Ltd., Tokyo, Japan [37,38]. This technique utilizes a DNA polymerase with strand-displacement activity, such as Bst and GspSSD-LF DNA polymerase, which removes the need for temperature cycling (like in PCR) to denature double-stranded DNA. The DNA polymerase in LAMP allows for isothermal amplification, meaning amplification can occur at a fixed temperature. This significantly simplifies the equipment needed to incubate the reaction and allows for operation without instruments or electricity. Additionally, both Bst and GspSSD-LF enzymes have reverse transcriptase (RT) functionality, allowing LAMP to detect both DNA and RNA targets.

### 2.1. LAMP Primers

**Figure 2:**
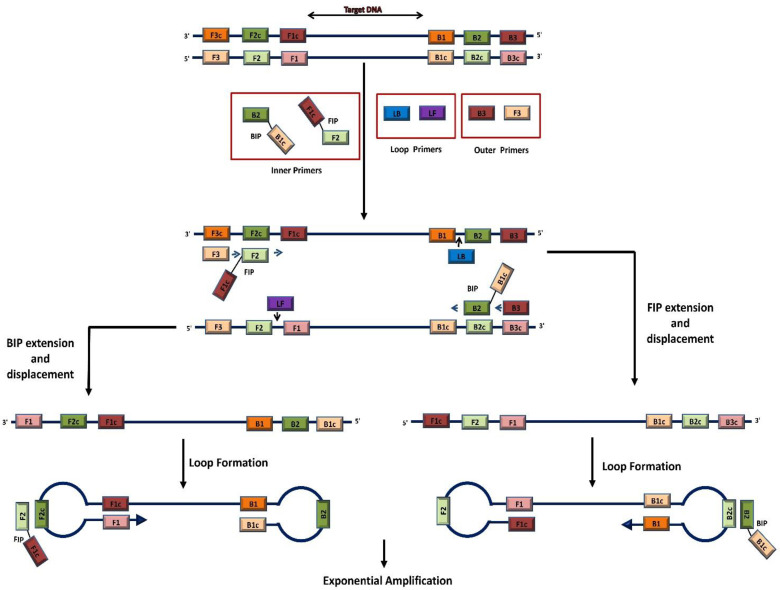
Schematic illustration of the loop-mediated isothermal amplification (LAMP) reaction and its underlying principle. LAMP utilizes four main primers (F3, FIP (F2 + F1c), B3, and BIP (B2 + B1c)) along with two additional loop primers (LF and LB) that recognize different sequences of the target nucleic acid. LAMP operates through a series of strand displacement and DNA synthesis events, producing large quantities of amplified DNA.

Like PCR, LAMP can amplify DNA using two primers—forward (F3) and reverse (B3)—that bind to opposite strands of the target DNA. However, LAMP typically uses four to six primers to increase the amplification rate significantly. These include forward and backward inner primers (FIP and BIP) and optional loop primers (LF and LB). FIP and BIP consist of a forward or backward internal sequence (F2 or B2) at the 3′ end and a sequence complementary to the F3 or B3 primer at the 5′ end, facilitating the formation of a stem–loop structure. The optional loop primers (LF and LB) are often added to further speed up amplification. These primers bind to loop structures formed during the LAMP process, enhancing the amplification of the target sequence (Figure 2). LAMP operates through a series of strand displacement and DNA synthesis events, producing large quantities of amplified DNA with high specificity and efficiency [22].

Various specialized online tools are available for LAMP primer design, such as Primer Explorer, Eiken Chemical Co., Ltd. (https://primerexplorer.jp/e/, accessed on 27 May 2024), and Optigene’s Primer Design Service (http://www.optigene.co.uk/products-primer-design-service/, accessed on 27 May 2024). Before designing LAMP primers, primer sites can be identified by the sequence alignment of available genomes to ensure that the primers target conserved regions of genetically related strains to minimize false negatives. The variability of sequences can be overcome, to a degree, with degenerate primers and/or primers that incorporate synthetic locked nucleic acids (LNAs) to enhance affinity [39].

Other factors that need to be considered are oligonucleotide length, G-C content, melting temperature, susceptibility to secondary structures, and susceptibility to primer-dimer formation.

Typically, F3 and B3 primers should be 18–20 nucleotides long, while FIP and BIP primers should be 38 to 42 nucleotides long. The G‒C content of the primer set needs to be optimized for the targeted regions (~300 nucleotides), ensuring the detection of short, conserved regions within the genome. LAMP oligonucleotides’ G‒C content typically ranges between 50% and 60%. The melting temperature (Tm) of the oligonucleotides can be predicted using the nearest neighbor method [40], aiming at approximately 60 °C to 65 °C for GC-rich regions and 55 °C to 60 °C for AT-rich regions [37,38].

To design a LAMP assay to detect a gene of interest, assay conditions, such as incubation temperature, reaction time, and amplicon detection method, must also be considered. The optimal incubation temperature successfully hybridizes the four primary LAMP primers (F3, B3, FIP, and BIP) with the target sequence. The optimal incubation temperature is usually determined by incubating the LAMP assay at various temperatures from 58 °C to 73 °C and selecting the incubation temperature that minimizes the threshold time Tt. Typically, the LAMP assay tolerates temperature variations and performs well within ±3 °C of the optimal incubation temperature [37,41,42,43,44,45,46,47,48].

### 2.2. LAMP Incubation

LAMP can be incubated with any standard thermal cycler operating at a fixed temperature. Since no thermal cycling is needed and LAMP tolerates a range of incubation temperatures well, incubation can also be carried out with simple means such as water baths and heat blocks without the need for precise thermal control, making LAMP suitable for use at locations with constrained resources [22].

Furthermore, LAMP incubation can be carried out entirely electricity-free with heating provided by an exothermic chemical reaction like in Meals Ready to Eat (MREs) [49], wherein a magnesium alloy interacts with salt water to produce heat. Temperature control is achieved with a phase change material (PCM) that undergoes a phase transition at approximately the LAMP incubation temperature. As we know from elementary physics, this provides precise temperature control so long as the two PCM’s phases coexist. Researchers have demonstrated the ability to maintain a LAMP incubation temperature of ~65 °C for nearly an hour at ambient temperatures as low as 10 °C and as high as 40 °C [50]. The ability to incubate the reaction electricity-free and detect amplification instrument-free opens the door for fully disposable, inexpensive molecular diagnostic devices that can be used for chicken virus detection at the point of need [51].

Numerous inexpensive incubators and readers are available when fluorescent detection is desired. A few examples include ESE-Quant TubeScanner (Qiagen Lake Constance GmbH, Stockach, Germany), Genie II (Optigene, Horsham, UK), and EzDx WeD (Hangzhou, China http://en.ezdxtech.com/, accessed on 3 June 2024). Excitation and detection can also be facilitated with smartphone flash and camera, respectively, with the added capabilities of communications and surveillance [47,51].

In summary, LAMP is (1) simple to execute; (2) rapid (less than an hour); and (3) can be carried out at a constant incubation temperature (around 65 °C) with simple equipment or no equipment at all.

### 2.3. LAMP Product Detection

**Figure 3:**
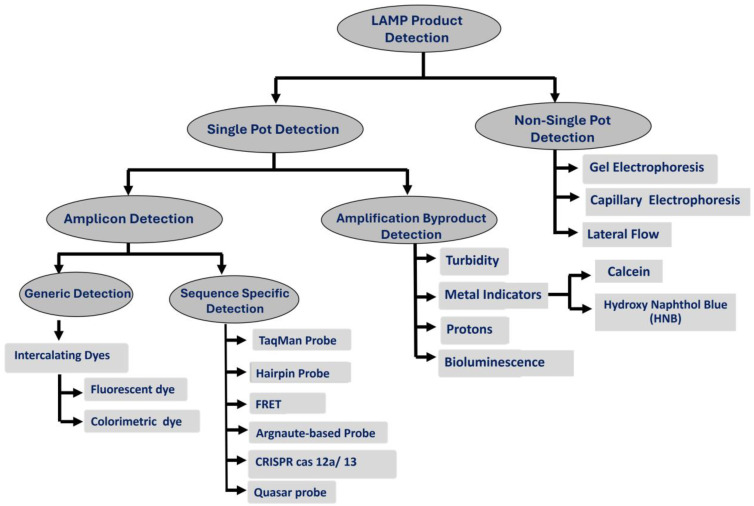
The various methods used for LAMP product detection.

LAMP is more efficient than PCR, increasing the number of amplicons by approximately 10^10^-fold in less than an hour, allowing for detecting a single target copy in a reaction volume. This large production of DNA amplicons and reaction byproducts offers numerous opportunities for both instrumented and instrumentation-free detection methods [51,52]. These detection methods can be broadly classified into single-pot and non-single-pot methods (Figure 3). Single-pot detection methods can be divided into direct amplicon and polymerase byproduct detection methods. Non-single-pot detection methods, which rely on amplicon detection, include gel and capillary electrophoresis and lateral flow. Direct amplicon detection methods can be further categorized into generic and sequence-specific methods. Some of these detection methods can be performed in real-time, enabling the quasi-quantification of templates based on the signal threshold time (Tt), while others are only suitable for endpoint detection. 

When adding dye or dyes to the LAMP reaction mix, several factors must be considered. An adequate amount of dye is necessary to produce a sufficiently bright signal for detection while maintaining a reasonable signal-to-noise ratio. The required amount partly depends on the sensitivity of the detector being used. However, using too much dye can have inhibitory effects on the reaction.

#### 2.3.1. Single-Pot Detection

A. Amplicon Detection

A.1. Generic Amplicon Detection

Non-sequence-specific LAMP amplicon detection methods often utilize intercalating dyes, like PCR. These dyes are self-quenched when free in solution but significantly increase their fluorescence emission intensity when they intercalate between the base pairs of double-stranded DNA (dsDNA) [53].

Some common fluorescent dyes include SYBR Green, Pico Green, Eva Green, and various SYTO dyes. These dyes are excited by UV light at wavelengths less than 500 nm and emit green visible light at wavelengths greater than 520 nm. The emission intensity can be significantly enhanced by the presence of dispersed metallic nanoparticles, a phenomenon known as the metal-enhanced fluorescence (MEF) effect [54,55].

Less common intercalating dyes operate competitively, having a greater affinity for dsDNA than for a color-suppressor, thus causing a color change upon binding to dsDNA. Another class of intercalating dyes, such as Leco Crystal Violet (LCV), does not require excitation and changes color from colorless to deep violet upon intercalation and oxidation [56]. These various dyes usually provide a bright signal and can be monitored either in real-time, enabling quantification, or at the endpoint for positive/negative results.

Generic, intercalating dyes keep assay costs low, reduce development time, and provide bright signals (and high sensitivity) because multiple dye molecules bind to a single amplicon. The specificity of these assays is provided by the LAMP primers, and amplification fidelity can be determined using melting curve analysis [57,58].

A.2. Specific Amplicon Detection

In most cases, LAMP amplifies DNA with high accuracy. However, unintended primer interactions can sometimes produce spurious amplicons. To prevent false positives, sequence-specific amplicon detection methods are used. Here are a few examples of such methods.

A.2.1. TaqMan Probe

A fluorescent reporter is attached to the 5′ end of the probe, and a fluorescent quencher is attached to the 3′ end. During the annealing step, when the probe oligo hybridizes to the target DNA, the probe–target hybrid becomes a substrate for the polymerase’s 5′ to 3′ exonuclease activity. This activity degrades the probe, releasing the fluorophore and resulting in increased fluorescence emission [57,59].

A.2.2. Hairpin Probe

A thermally stabilized hairpin probe, typically using locked nucleic acids (LNAs), has a fluorophore at one end and a quencher at the other. When the probe hybridizes with the amplicon, usually in the loop region, the distance between the fluorophore and quencher increases, leading to light emission [60].

A.2.3. Fluorescent Resonance Energy Transfer (FRET)

The hybridization probe consists of two oligonucleotides, one potentially serving as a primer, complementary to two adjacent regions of the target template. One oligonucleotide contains a donor, while the other contains an acceptor. When hybridized to the target, the donor is brought close to the acceptor. The energy emitted by the donor is absorbed by the acceptor, resulting in a significant increase in emission intensity [61,62].

A.2.4. Argonaute-Based Probes

Argonaute-Based Probes include, e.g., *Pyrococcus furiosus* Argonaute (*PfAgo*). LAMP amplification products are heated to the appropriate incubation temperature for the Argonaute protein (e.g., 95 °C for *PfAgo*). *PfAgo*, guided by two guides with sequences complementary to the amplicon, cleaves a fragment of the amplicon, which then serves as a guide for *PfAgo*. This guide-*PfAgo* complex subsequently cleaves a sequence-complementary oligo probe, separating the quencher from the emitter. Agos activity is independent of motifs like the proto-spacer adjacent motif (PAM) or proto-spacer flanking site (PFS) in target sequences. Agos uses short guide DNA (gDNA), which is easy to design, cost-effective to synthesize, and less prone to degradation during use than Cas RNA guides. Since the Ago is a turnover enzyme, it cleaves complementary oligo probes, amplifying the signal. Unlike Cas 12a and Cas 13, which, once activated, have trans activity and cleave probes indiscriminately, *PfAgo* guided by amplicon fragment cleaves selectively complementary probes with single nucleotide precision, enabling multiplexing. Most recent studies have utilized thermophilic pAgos (prokaryotic Agos), which operates at high temperatures ranging from 66 to 95 °C. This temperature requirement likely accounts for the limited development of Ago-based systems for diagnostic applications [35,63,64,65].

A.2.5. CRISPR Cas 12a/13

RNA-guided CRISPR Cas 12a/Cas 13 are activated upon binding to a complementary segment of the amplicon and, through trans activity, indiscriminately cleave oligo/RNA probes that are functionalized with a fluorophore and a quencher to produce a signal. CRISPR/Cas-based sensors have some limitations, including the requirement for a PAM or PFS in the target sequence and their dependence on RNA guides, which are expensive and easily degradable, resulting in low adaptability. Additionally, since Cas 12 a/13 cleaves any proximate DNA/RNA indiscriminately, detecting multiple targets simultaneously with these sensors in a single pot is technically challenging [66,67,68,69].

A.2.6. Quasar probes

In QUASR detection, one of the loop primers (LF or LB) or inner primers (FIP or BIP) is labeled with a fluorophore. The reaction mixture also includes a short probe labeled with a dark quencher at its 3′ end, which is complementary to 7–13 bases at the 5′ end of the dye-labeled primer. The quencher probe is present in slight excess relative to the labeled primer. It has a melting temperature (Tm) less than 10 °C below the LAMP reaction temperature, ensuring the probe and primer remain dissociated during LAMP amplification. After incubation, dark quenching of fluorescent primers occurs in the absence of amplicons after the reaction mix has cooled to ambient temperature. In the presence of amplicons, the probe cannot hybridize with the quencher, resulting in bright fluorescence emission [33].

All methods for specific amplicon detection, except for the CRISPR-based approach, are amenable to multiplexing with multi-color probes. The primary challenge in multiplex detection lies in achieving analytical sensitivity comparable to single-plex assays. To accomplish this, it is essential to use well-designed primers and probes in the correct ratios and to optimize experimental conditions to improve the amplification efficiency. This challenge extends the development time for multiplex assays [33,35,70,71]. While the multiplexed assay can simultaneously detect multiple targets, the probability of having multiple targets in a single sample is low.

B. Detection of amplification byproducts

Since LAMP generates substantial amounts of DNA along with byproducts like magnesium pyrophosphate precipitate and protons, the presence of polymerase can be detected through these byproducts. Though non-specific, these methods allow for visual detection of whether amplification took place. A few byproduct detection methods can be used simultaneously with direct amplicon detection methods with minimal or no interference.

To understand byproduct detection methods, it is instructive to examine the chemistry of the polymerase process. During extension, the polymerase incorporates deoxyribonucleoside triphosphates (dNTPs) to complement the ssDNA template by linking the 5′ alpha phosphate group to the OH group of the extending sequence (*Dn*) in the presence of Mg^2+^ ions. The synthesis reaction byproducts include pyrophosphate (PPi, P_2_O_7_-) formed from the dNTP’s beta and gamma phosphates and a proton released from the primer’s 3′-OH group [72,73].

$$D_{n}+dNTP\overset{enzyme,\;Mg^{2+}}{\to }D_{n+1}+PP_{i}+H^{+}$$

The pyrophosphate interacts with metal ions to form low-solubility metal-pyrophosphate compounds, such as Mg_2_O_7_P_2_, which precipitate from the solution. The polymerase process can be monitored in real-time or at the endpoint by observing, among other things, changes in solution turbidity due to metal-pyrophosphate precipitation, pH variations due to proton release, fluctuations in Mg^2+^ concentration, and the charge of the synthesized DNA [74].

B.1. Turbidity-based detection

The substantial quantity of DNA, up to 400 μg/mL, produced during LAMP results in a significant amount of white magnesium pyrophosphate precipitate, which increases the solution’s absorbance. This absorbance can be tracked in real-time with a spectrophotometer (at approximately 400 nm) or visually observed at the endpoint. However, the visual signal is faint, making the assessment of a positive or negative test subjective [75,76].

B.2. Metal indicators

Metal indicators are an attractive alternative to monitoring turbidity.

B.2.1. Calcein (Fluorexon, C_30_H_26_N_2_O_13_)

Calcein is a fluorescent dye with excitation/emission wavelengths of 495 nm/515 nm. When calcein complexes with manganous ions (Mn^2+^), its fluorescence is quenched, causing the solution to appear orange. During LAMP reaction, Mn^2+^ ions preferentially bind to pyrophosphate ions (P_2_O_7_^4−^) and precipitate, leaving free (green) fluorescing calcein. The fluorescence intensity of calcein is further enhanced when it binds with Mg^2+^.

$$Mn^{2+}•Calcein\to Mg^{2+}•Calcein+Mn_{2}P_{2}O_{7}\left(s\right)↓$$

Fluorescence can be excited by daylight, resulting in weak emission, or with a UV light source at 365 nm, resulting in strong emission. The concentration of Mn^2+^ in the reaction mix must be carefully optimized. High Mn^2+^ concentrations can inhibit polymerase activity [52,77,78,79].

B.2.2. Hydroxy naphthol blue (HNB)

HNB is another metal indicator frequently used to detect amplification byproducts. Adding 120 µM HNB to the LAMP reaction mix does not significantly inhibit amplification efficiency. During the polymerase reaction, as Mg^2+^ ions are depleted due to their binding with pyrophosphate and subsequent precipitation, HNB changes color from violet to sky blue.

$$Mg^{2+}•HNB\to HNB+Mg_{2}P_{2}O_{7}\left(s\right)↓$$

This change can be monitored in real-time using a spectrophotometer at 650 nm [52,77,78,79].

B.3. Protons

Proton release from polymerase is utilized by solid-state sequencers such as the Ion Torrent. The LAMP process generates many amplicons and protons, sufficient to cause a detectable pH shift from alkaline to acidic in a weakly buffered reaction mix (e.g., 26 µM Tris). This pH change can be monitored in real-time with a pH meter or field effect transistor or visually observed using various pH indicator dyes, such as phenol red, which changes color from pink (alkaline conditions, prior to and in the absence of amplification) to yellow (acidic conditions, positive test). One challenge of these assays is that the pH of the reaction mix can be affected by factors unrelated to polymerase activity. For example, adding unpurified samples can cause pH and color changes all by itself. Sample effect on pH has been observed, among other things, in saliva sample tests for SARS-CoV-2.

Additionally, extending the incubation time to 60 min resulted in some color changes, which we attribute to non-template amplification. This conclusion is supported by intermediate color changes observed in some control reactions [58,80].

B.4. Bioluminescence

When the enzyme ATP sulfurylase (APS) is added to the reaction mix, it converts the polymerase byproduct inorganic pyrophosphate (PPi) into ATP.

$$PPi+APS\to ATP+SO_{4}^{2-}$$

The ATP then fuels luciferin to produce visible light, like the bioluminescence in fireflies.

$$luciferin+ATP+O_{2}\to Oxyluciferin+AMP+PPi+CO_{2}+light$$

This method has the advantage of not requiring excitation and being free of any background. However, the light must be continuously monitored with a photodetector, a camera, or a smartphone camera programmed for a long exposure time. At the early stages of amplification, ATP production is low, and no detectable light is emitted. As the polymerase rate increases, ATP production and light intensity increase and eventually peak. Once the polymerase reaction saturates, light emission ceases. The time interval between the start of incubation, until detectable light appears, correlates logarithmically with the number of templates and can be used for quantification, similar to the threshold time (Tt) used with fluorescent dyes [42,43,47].

#### 2.3.2. Post-Amplification Detection

Earlier methods of amplicon detection, which are still occasionally used, include gel electrophoresis, capillary electrophoresis (CE), and lateral flow strips [52]. These methods often require opening the reaction tube and transferring amplicon-rich reaction products, posing a significant risk of carryover contamination.

Gel Electrophoresis: LAMP amplicons form stem-loops with inverted repeats and cauliflower-like structures, resulting in a ladder-like electropherogram rather than a single band, as is common with PCR products.

Lateral Flow: For detection with lateral flow strips (LFSs), LAMP primers are functionalized with small molecules such as biotin and/or digoxigenin (dig). The LAMP reaction products are discharged onto the sample pad of a porous nitrocellulose strip equipped with at least two capture lines; one (test) line captures the amplicons, and the other (control) line captures the labels. The sample migrates up the strip by capillarity; goes through a conjugate pad; hydrates dry-stored reporters, which then bind to the amplicons through, for instance, dig–anti-dig affinity; passes through the test and control lines; and into the absorption pad, which then acts like a “pump”. The streptavidin-functionalized test line captures the labeled amplicons. The control line is typically functionalized with mouse IgG and serves to monitor test integrity.

Reporters are often gold nanoparticles or colored latex beads that produce color change at the test and control lines visible to the naked eye. LFSs, also known as rapid tests, have low sensitivity but are inexpensive and easy to use. To increase sensitivity, reporter particles can be replaced with fluorophores, quantum dots, up-converting phosphor particles (UCPs), and enzymes, all of which require excitation and a reader.

Most natural materials, including nitrocellulose, self-(auto) fluoresce. Hence, careful optimization is needed to minimize background emission when fluorescent reporters are used. In contrast, UCPs have the advantage of emitting at wavelengths smaller than the exciting light (anti-Stokes shift)—something that is not exhibited by natural materials—thus, eliminating background.

The reporter can also be an enzyme (like ELISA) that produces a color change or CRISPR Cas 12a that is activated upon binding to the amplicon and cleaves a quenched oligo probe. Both lead to signal amplification. Many other variants are possible.

In most cases, LAMP amplicons are detected directly in the LAMP reaction chamber. The use of external detection, such as electrophoresis and LFSs, adds complexity and is justified only in special applications. Table 1 summarizes the working principles, advantages and disadvantages of various LAMP product detection methods.

Although LAMP offers several important advantages over PCR, like rapidity, simplicity, and high sensitivity, it comes with disadvantages. LAMP requires four to six primers to recognize distinct regions of the target templates. The design of these primers is more complex and time-consuming than the design of PCR primers. LAMP is more susceptible to non-specific amplification than PCR. Careful optimization is needed to minimize false positives. LAMP is more tolerant of contaminants in the sample than PCR but may still be inhibited by certain components in the sample when non-purified samples are used. As template concentration decreases, the variance in LAMP threshold time increases, complicating quantification. The sequencing of LAMP amplicons is more challenging than that of PCR amplicons [21,81].

## 3. Two-Stage Penn-RAMP Technique

Infectious diseases in humans, animals, and plants often present non-specific symptoms but may require diverse control strategies. While most molecular diagnostic devices are single-plex or, at most, have limited multiplexing capabilities, precision disease control requires the co-detection of multiple co-endemic pathogens. Low-level multiplexing can be achieved with multicolor probes that require expensive readers and are typically limited to five channels (colors) or with lateral flow strips equipped with multiple test lines, each functionalized to capture a specific amplicon. To achieve high-level multiplexing, one can aliquot the sample into multiple LAMP reaction chambers, each pre-storing a primer set specific to a single pathogen or primer sets for a group of pathogens. Since this strategy requires sample dilution, it may adversely impact assay sensitivity.

**Figure 4:**
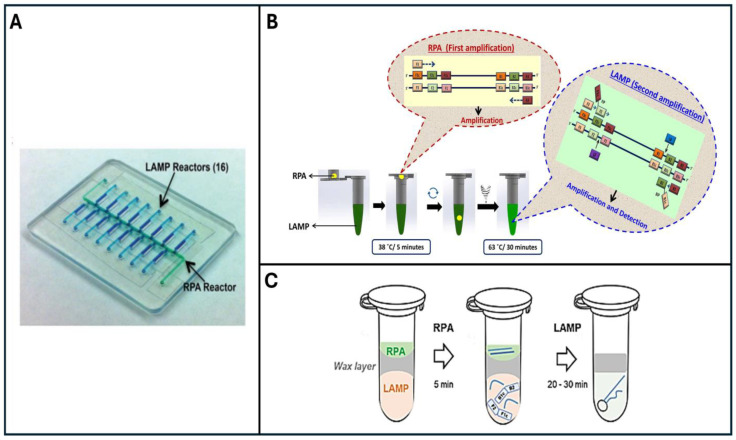
Two-stage Penn-RAMP closed system (adapted from [34,36,82]). (**A**) A microfluidic device designed for Penn-RAMP’s amplification in a sealed microfluidic chip, comprising a central multiplex RPA reactor (green) and 16 branching LAMP reactors (blue) for targeted samples and controls. (**B**) The operation of the closed-tube Penn-RAMP assay. (**C**) The PEN-RAMP tube includes two compartments divided by a thermally removable barrier.

The Penn-RAMP two-stage isothermal amplification method [34] remedies this situation. Penn RAMP comprises two stages of isothermal amplification. The first stage is RPA (recombinase polymerase amplification), which includes primers for all targets of interest. Since RPA requires only two primers per template, it is amenable to a greater level of multiplexing than LAMP. In the first stage, the templates of interest are amplified at 38–42 °C for a short time, enough to produce large numbers of templates for the second stage but not enough to produce detectable signals. The first stage amplicons are then aliquoted into multiple LAMP reaction chambers, each specialized to detect a single target or a group of targets. Therefore, this method enables high-level multiplexing (as many as 16 targets were successfully demonstrated, Figure 4A) without a loss of sensitivity.

The two-stage process can be carried out in a self-actuated chiplet [Unpublished data]. Typically, one of the second-stage LAMP reaction chambers is designated as positive control and detects a housekeeping gene. Another second-stage reaction chamber is designated as negative (primer-free) control to guard against the production of detectable signals during first-stage amplification.

Serendipitously, Penn-RAMP provides much better sensitivity than LAMP alone, even in a single-plex format. In single plex, Penn-RAMP can be implemented in a single pot with the RPA reaction taking place in the tube’s lid (as a hanging drop) and the LAMP reaction inside the tube’s body [36,82] (Figure 4B,C). The tube is first incubated briefly at the RPA incubation temperature for 5–10 min. Next, the tube is flipped upside down a few times to mix the RPA amplicons with the LAMP reaction mix. Then, the tube is incubated at 65 °C for LAMP amplification. This allows for the detection of amplification products without opening the tube [36].

Alternatively, single-plex Penn-RAMP can be carried out in a single pot with two compartments separated by a thermally removable barrier. The first stage of RAMP (RPA) occurs above the barrier at the RPA incubation temperature. The barrier melts away as the pot is heated to the LAMP incubation temperature, allowing the RPA reaction volume to mix with the pre-stored LAMP buffer in the lower compartment. Thus, facilitating second-stage amplification [82]. Penn-RAMP assays concord with the gold standard quantitative PCR (qPCR) assay, exhibiting, however, a limit of detection (LOD) 10 times superior to both LAMP and qPCR (Table 2).

The Penn-RAMP method suffers from the same disadvantages as LAMP, although it is more contaminant-tolerant than standalone LAMP. The volume of RPA products added to the LAMP mix is restricted to avoid inhibiting LAMP. This typically is not a concern in high level multiplexed assays. Despite these disadvantages, LAMP and Penn-RAMP offer many benefits for applications at the point of need.

## 4. Detection of Respiratory Tract Viruses in Broiler Chickens with LAMP

**Figure 5:**
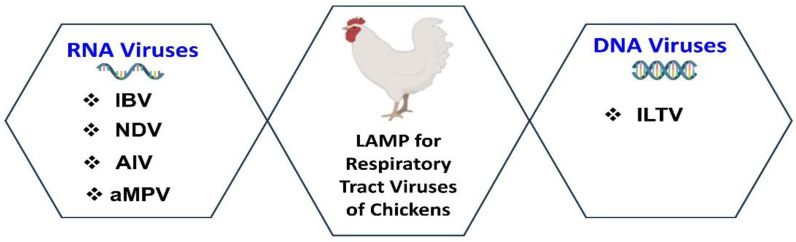
Respiratory tract viruses identified with LAMP and Penn-RAMP in chickens: DNA virus (infectious laryngotracheitis virus (ILTV)) and RNA viruses (Newcastle disease virus (ND), infectious bronchitis virus (IBV), avian influenza virus (AIV), and avian metapneumovirus (aMPV)).

The respiratory tract viruses detected in chickens using LAMP or Penn-RAMP are discussed in our review and summarized in Figure 5. These include the DNA virus infectious laryngotracheitis virus (ILTV) and the RNA viruses Newcastle disease virus (NDV), infectious bronchitis virus (IBV), avian influenza virus (AIV), and avian metapneumovirus (aMPV).

### 4.1. DNA Virus

#### Infectious Laryngotracheitis Virus (ILTV)

The infectious laryngotracheitis virus ILTV is a DNA virus classified under the *Gallid alphaherpesvirus* 1 (GaHV-1) species within the *Alphaherpesvirinae* subfamily of the *Herpesviridae* family. ILTV induces respiratory difficulties and contributes to substantial losses in production, including reduced egg output, inefficient feed conversion, elevated mortality rates, and heightened vulnerability to opportunistic respiratory infections [83,84,85,86].

An ILTV-LAMP assay, which relies on electrophoresis, has been developed as a reliable alternative to PCR for the accurate molecular diagnosis of ILTV infections. This method has demonstrated a sensitivity ten times greater than that of the PCR assay [87]. Unfortunately, this detection technique requires two steps and exposes reaction products to the ambient environment, leading to carry-over contamination and false positives. The effective performance of the ILTV-LAMP assay has been validated across a relatively broad temperature range, ranging from 62 to 65 °C, rendering this assay a practical point of need diagnosis where strict temperature control may be challenging. Yu et al. [88] introduced a modified closed-pot LAMP assay employing a real-time turbidity meter and calcine for reaction detection, mitigating the risk of cross-contamination and achieving 100-fold better sensitivity than PCR.

**Figure 6:**
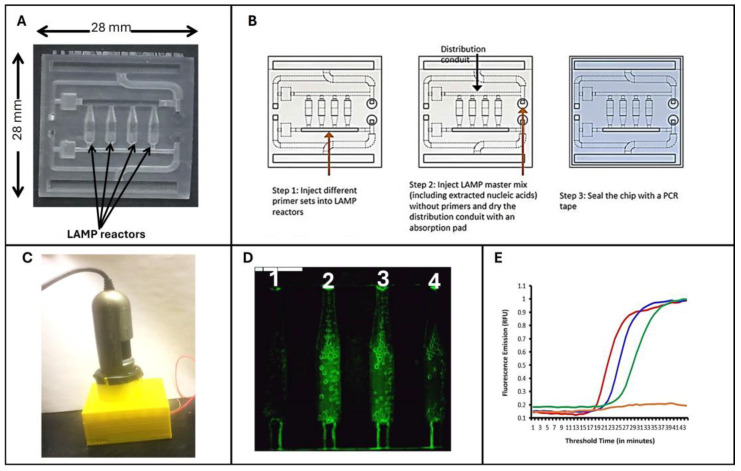
(**A**) A 3D-printed microfluidic chip with four multifunctional reactors for real-time LAMP reactions. (**B**) Schematic representation for the steps of possible co-detection of three different pathogens in a single sample and a control. (**C**) The portable fluorescent microscope monitors fluorescent emission from the microfluidic chip. (**D**) Image of fluorescing LAMP reactors. (**E**) Real-time amplification curves of microfluidic chip-based LAMP assays [89].

More recently, Eltholoth et al. [89] developed an ILTV-fluorescence-based LAMP assay that can be carried out with either standard laboratory equipment or integrated into a microfluidic device (Figure 6). This multiplexed microfluidic chip contains four reaction chambers. One chamber serves as a negative (no-primer) control. The remaining three reaction chambers co-detect three different pathogens by self-aliquoting a single sample into multiple chambers, each customized to amplify a specific target. The chip mates an affordable, portable, homemade processor that controls temperature and detects fluorescence with a USB camera connected to a laptop. The USB camera captures the excited fluorescent emissions from all reaction chambers once every minute and transmits the data to a portable device such as a laptop computer. Alternatively, the chip can be linked to a smartphone for data analysis and test results reporting, as previously described [24,27]. The chip-based LAMP method exhibited comparable analytical sensitivity to that of qPCR. In another effort, Eltholoth et al. [36] employed a single-plex Penn-RAMP for ILTV detection with approximately tenfold better sensitivity than the standalone LAMP assay.

### 4.2. RNA Viruses

#### 4.2.1. Infectious Bronchitis Virus (IBV)

The infectious bronchitis virus IBV is a major respiratory disease affecting chickens and has significant economic consequences for the poultry industry. It is caused by a single-stranded RNA coronavirus. Clinical signs of the disease include respiratory manifestations and a decrease in egg quality and production. IBV exists in various serotypes and genotypes worldwide. Recently, a new classification system for IBV was introduced using phylogenetic analysis of the S1 gene. This classification identified six genotypes, which were further divided into 32 lineages. Over the past decade, there has been an increase in reported IBV strains that cause nephritis [90,91,92,93,94,95,96,97].

Recently, researchers have shifted towards using RT-LAMP assays for IBV [20,98,99]. These assays offer several advantages over PCR, including speed, constant temperature, and abundant amplicons, allowing these tests to be performed with simple instruments. The afore-described assays use lateral flow strips, gel electrophoresis, and the naked eye for amplicon detection and are not amenable to quantification. Furthermore, opening a tube rich in amplicons to enable lateral flow-based detection or electrophoresis risks carrying over contamination of the test site, potentially rendering subsequent tests false positives.

El-Tholoth et al. [21] developed a semiquantitative, closed-tube, single-step, real-time RT-LAMP assay for IBV detection. The LOD (limit of detection) of the RT-LAMP assay is comparable to that of the RT‒PCR assay [21] (~1 EID_50_/mL). Previously developed IBV-RT-LAMP assays reported a LOD of 200 EID_50_/mL [20,98]. The increased analytical sensitivity observed in the closed tube assay may be attributed to the LAMP amplicon detection method. Earlier assays [20,98] relied on visual inspection or gel electrophoresis, while El-Tholoth et al. [21] used real-time fluorescence monitoring. Additionally, the use of loop primers (LB and LF) [21], which were absent in earlier assays [20,98], improved amplification efficiency and enhanced sensitivity. Since early-stage IBV infections in chicken respiratory tissues and secretions typically have IBV EID_50_ levels exceeding 10^2^ copies/μL, the detection limit of the LAMP assay is adequate for virus detection without sample concentration. Additionally, El-Tholoth et al. [36] utilized the single-plex Penn-RAMP for IBV detection, which showed approximately 10-fold better sensitivity than the standalone LAMP assay with the same template.

#### 4.2.2. Avian Influenza Virus (AIV)

Avian influenza (AI), commonly referred to as “bird flu”, is a contagious disease that affects poultry populations, leading to a significant mortality rate and resulting in reduced production, economic losses, and restrictions on the movement of birds. Control of this disease and eradicating outbreaks is costly as it requires the destruction of many birds. Influenza virus is an RNA virus belonging to the *Orthomyxoviridae* family and is categorized into seven genera: *Influenza virus A*, *Influenza virus B*, *Influenza virus C*, *Influenza virus D*, *Thogotovirus*, *Isavirus*, and *Quarajavirus*. There have been sporadic reports of zoonotic infections in which the virus is transmitted from birds to farm animals and from animals to humans, resulting in fatalities of farm animals. These zoonotic infections are associated with specific subtypes of avian influenza viruses, such as H1N1, H2N2, H5N1, H7N7, and H7N9 [1,3,100,101,102]. The various subtypes of the avian influenza (AI) virus are identified by the antigens found on the surface of the influenza A virus. To date, 16 hemagglutinin (HA) subtypes and 9 neuraminidase (NA) subtypes have been identified. However, more recent research revealed the existence of new HA subtypes, totaling 18, and NA subtypes, totaling 11. These new subtypes were discovered in bats [103,104].

H5, H7, and H9 are the most identified subtypes of avian influenza viruses (AIVs). H5 and H7 subtypes are classified as highly pathogenic AIVs (HPAI), which result in severe illness and high mortality rates. The H9 subtype, on the other hand, is categorized as a low pathogenic AIV (LPAI), causing mild respiratory symptoms and a notable decrease in egg production among laying chickens. Co-infection involving the H9 subtype alongside other pathogens can exacerbate respiratory signs and mortality rates. It is crucial to quickly differentiate between the H5, H7, and H9 subtypes to implement appropriate vaccination programs that target the circulating subtype(s) [101,102].

In numerous studies, the LAMP method has proven effective in accurately detecting the widespread avian influenza virus subtype H5N1 [105,106,107,108]. Furthermore, the LAMP method has demonstrated promising sensitivity in detecting avian influenza virus subtype H7, achieving a level of sensitivity of 0.01 PFU/reaction, which is 100 times better than that of RT‒PCR [109]. This finding is consistent with previous research on LAMP diagnosis for avian influenza virus subtype H5 [105]. The detection limit of the LAMP method for low pathogenic avian influenza (LPAI) H7 subtypes is comparable to that of highly pathogenic avian influenza (HPAI) [109]. For subtype H9, the detection limit of the LAMP method is 10 copies, showing tenfold greater sensitivity than RT‒PCR [18]. Additionally, an RT-LAMP method developed specifically for avian influenza subtype H7N9 exhibited high specificity and sensitivity of up to 50 copies/reaction, making it suitable for direct RT-LAMP without the need for additional nucleic acid extraction procedures [110].

#### 4.2.3. Newcastle Disease Virus (NDV)

*Avian paramyxovirus* 1 (APMV-1), more commonly referred to as Newcastle disease virus (NDV), is a pathogen that leads to Newcastle disease (ND), a highly contagious disease affecting avian populations globally. This virulent strain is responsible for significant economic losses within the poultry sector. Initially identified in 1926 in Newcastle, England, this virus is classified under the *Orthoavulavirus* genus, *Avulavirinae* subfamily, and *Paramyxoviridae* family (revised nomenclature for Newcastle disease virus and updated unified phylogenetic classification system [111,112,113]).

APMV-1 comprises two distinct groups, referred to as class I and class II. Most class I viruses are characterized by low virulence and are typically identified in wild birds, such as waterfowl and shorebirds, as well as in live poultry. However, class II viruses are highly virulent and have been detected in poultry, pets, and wild birds. Class II viruses are further classified into 21 genotypes (genotyped I–XXI) and 5 pathotypes (viscerotropic velogenic, neurotropic velogenic, mesogenic, lentogenic, and asymptomatic) based on their clinical manifestations and pathological characteristics [114,115,116,117,118].

Pham et al. [17] designed a LAMP assay targeting the F gene, which takes approximately 2 h to complete. The reaction involves outer and inner primers (without loop primers) and requires incubation at 65 °C for 120 min, followed by heating at 80 °C for 10 min to stop the reaction. Gel electrophoresis is used to detect the LAMP amplicons. The assay demonstrated an analytical sensitivity of 9 × 10^4^ copies/reaction. Subsequently, Li et al. [19] developed a one-step reverse transcription (RT)-LAMP assay that integrates amplification with reverse transcription into a single reaction tube. This method was designed for detecting the virus in various infected tissues such as the brain, lung, and intestine. Compared to the assay by Pham et al. [17], Li et al.’s assay is faster and more sensitive. It incorporates loop primers and employs colorimetric methods to visualize amplified DNA products, making it particularly suitable for laboratories with limited equipment. In a related development, Kirunda et al. [119] reported an alternative, cost-effective approach to RT-LAMP for detecting Newcastle disease virus (NDV) using less invasive samples like cloacal and oropharyngeal swabs. Their assay utilizes turbidity for visualization of DNA amplification, allowing for detection either by the naked eye or in real-time using a turbidimeter. Recently, Song et al. [120] developed NDV-Common-LAMP and NDV-Patho-LAMP assays, demonstrating specificity and sensitivity surpassing previous reports, with detection limits 10 to 10,000 times greater than those of earlier RT-LAMP assays. An assay for visually detecting both IBV and NDV via LAMP and lateral flow dipstick, with a limit of detection of 5 genome copies/reaction, was developed by Wu et al. [99].

#### 4.2.4. Avian Metapneumovirus (aMPV)

Avian metapneumovirus (aMPV) is a widespread pathogen found in poultry and various avian species, including wild birds [121]. It can cause respiratory illnesses and result in economic losses for the poultry industry [122,123]. aMPV is an RNA virus that belongs to the *Paramyxoviridae* family, specifically the *Pneumovirinae* subfamily [124]. The respiratory symptoms in chickens infected solely with aMPV are typically less pronounced and milder [125]. aMPV infections in chickens often worsen due to bacterial pathogens or other respiratory viruses [126,127,128].

Cea et al. [129] established a method combining RT-LAMP and colorimetric detection using DNA nanoprobes to detect aMPV in pharyngeal swabs and tracheal tissue samples. This system allows the virus to be detected in approximately 60 min through visible color changes observable with the naked eye. The assay they developed resulted in a final plasmonic biosensor that is straightforward to use, even in resource-constrained settings and has been demonstrated to be rapid, reliable, and precise. Their assay exhibited 100% specificity and 87.88% sensitivity compared with PCR. Moreover, comparing the LOD of their assay (50 copies per reaction) with that of fluorescent-based LAMP, they found that the fluorescence-based LAMP procedure has a sensitivity that is 10 times lower (LOD > 500 copies/reaction).

Table 3 provides an overview of the LAMP and Penn-RAMP assays for respiratory tract viruses in chickens, along with the detection limit for each assay.

## 5. Conclusions and Future Directions

This review summarizes the principles of the isothermal amplification assays, LAMP and Penn-RAMP, along with various methods for detecting LAMP products. These assays do not require thermal cycling and produce many amplicons, allowing them to be performed with simple, inexpensive instruments or even without any instruments by using an exothermic chemical reaction for heating and colorimetric dye for detection. Despite their simplicity, their performance rivals and sometimes even exceeds that of conventional laboratory-based methods such as PCR.

LAMP and Penn-RAMP were developed to detect respiratory tract pathogens in poultry, such as the DNA virus infectious laryngotracheitis virus (ILTV) and the RNA viruses Newcastle disease virus (NDV), infectious bronchitis virus (IBV), avian influenza virus (AIV), and avian metapneumovirus (aMPV). The main advantage of LAMP and Penn-RAMP is their suitability for on-farm use, enabling real-time disease detection. Rapid identification of these viruses would facilitate the implementation of appropriate control measures, reducing the number of infected animals and minimizing production losses. Conventional laboratory assays, typically conducted in centralized laboratories, are available to confirm the presence of respiratory viruses [7,8,9,10,11,12,13,14,15,16]. However, these diagnostic techniques require shipping samples to distant laboratories, which wastes precious time and may not be feasible in resource-limited settings.

In cases where avian viruses causing respiratory infections exhibit similar clinical signs, multiplexed assays capable of detecting multiple pathogens simultaneously are needed. Penn-RAMP, which combines RPA and LAMP, addresses this need by enabling the codetection of as many as 16 different pathogens or more and significantly enhancing sensitivity compared to LAMP, qPCR, and RPA when used individually.

LAMP and Penn-RAMP hold significant promise for clinical molecular diagnosis and virus surveillance in developing nations, particularly for respiratory diseases in chickens, without requiring advanced equipment or highly trained personnel. The reactions for both LAMP and Penn-RAMP can be adapted for use in microfluidic formats. These microfluidic chips offer advantages such as efficient operation, portability, sample containment, and user-friendly design, making them accessible to non-experts.

Based on this review, it is evident that developing multiplexed LAMP or Penn-RAMP strategies for detecting coinfections of respiratory tract viruses in chickens is highly important. Combining a simple nucleic acid extraction technique with LAMP and Penn-RAMP reactions could provide an effective screening test for combating pathogenic avian viruses. Further studies are essential to improve the ability of LAMP and Penn-RAMP assays to distinguish field virulent virus strains from vaccine strains, which is necessary for selecting appropriate control measures.

## Figures and Tables

**Table 1:** LAMP product detection methods.

	Technique	Working Principle	Advantages	Disadvantages	References
**1**	**Intercalating Dyes**	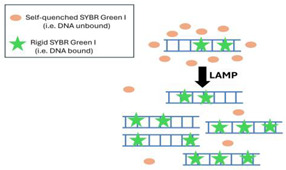	Sensitive, strong signal. Quantitative. Low cost. Single pot. Rapid implementation.	Non-specific. Requires excitation source and a reader. Considerable background. Susceptible to photobleaching. Negatively affect amplification efficiency if used in excess.	[53,54,57]
**2**	**TaqMan Probe**	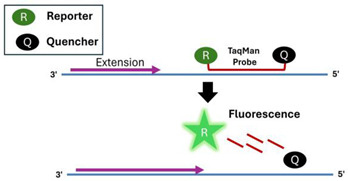	Highly specific. Quantitative. Single pot. Amenable to multiplexing. Low background.	Long development time. Non-generic. Requires excitation and a reader. Cost. Moderate sensitivity. Susceptible to photobleaching. Optimally restricted to single-strand regions such as the loop region.	[57,59]
**3**	**Hairpin Probe**	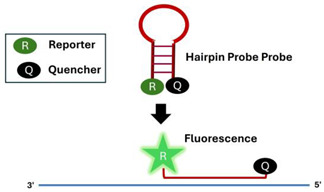	Highly specific. Quantitative. Single pot. Amenable for multiplexing. Low background.	Development time. Non-generic. Requires an excitation source and a reader. Cost. Moderate sensitivity. Susceptible to photobleaching. Thermal stability. Probes often need to include LNA. Optimally restricted to single-strand regions such as the loop region.	[60]
**4**	**Fluorescent Resonance Energy Transfer** **(FRET)**	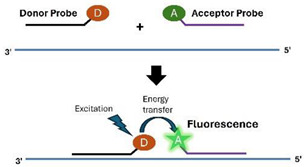	Highly specific. Quantitative. Single pot. Amenable for multiplexing. Low background.	Complex design. Non-generic. Requires excitation source and a reader Cost. Moderate sensitivity. Moderate emission. Susceptible to photobleaching. Optimally restricted to single-strand regions such as the loop region.	[61,62].
**5**	**Argonaute (*PfAgo*)-Based Signal Amplification**	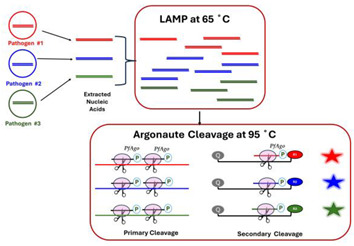	Highly specific. Quantitative. Single pot. Amenable for multiplexing. Low background. Signal amplification Inexpensive and stable oligo DNA guides.	Development time. Non-generic. Requires an excitation source and a reader. Cost. Susceptible to photobleaching Requires high reaction temperatures (66 °C for TtAgo, 95 °C for *PfAgo*).	[35,63,64,65]
**6**	**CRISPR Cas 12a/13**	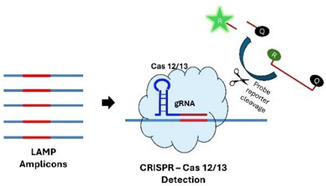	Highly specific. Quantitative. Signal amplification Active at room temperature (25–42 °C). Single pot (only with thermophilic Cas such as AsCas12a).	Complex design. Non-generic. Requires an excitation source and a reader. Cost. Temperature incompatibility when the common Cas12a (Cpf1) is used. Expensive, unstable RNA guides. Not amenable to multiplexing.	[66,67,68,69]
**7**	**Quasar Probes**	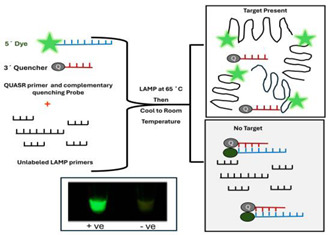	Specific. Sensitive. Quantitative. Single pot. Low background. No need for a reader when single plex. Amenable to multiplexed detection.	Complex design. Non-generic. Requires reader for real-time and/or multiplexed detection. Cost. Susceptible to photobleaching.	[33]
**8**	**Turbidity-Based Detection**	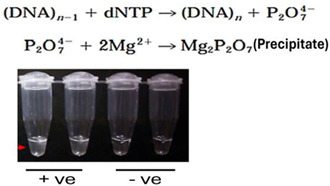	Naked eye endpoint detection Real-time detection with turbidimeter. Single pot. Simple. Low cost.	Non-specific. Low sensitivity. Not amenable to multiplexing. Subjective when detected by eye.	[75,76]
**9**	**Calcein (Fluorexon, C_30_H_26_N_2_O_13_)**	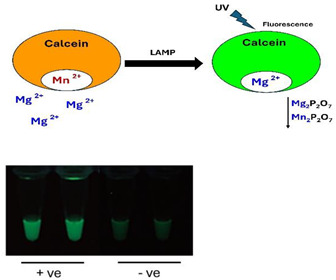	Sensitive Naked eye, endpoint detection. Single pot. Real-time monitoring. Simple. Low cost.	Non-specific Inhibitory effect of Mn^2+^ ions. Not amenable to multiplexing. Subjective endpoint naked eye reading.	[52,77,78,79]
**10**	**HNB (Hydroxy Naphthol Blue)**	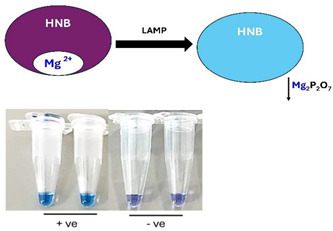	Endpoint, naked eye detection. Single pot. Simple. Low cost.	Non-specific. Low sensitivity. Not amenable to multiplexing. Not quantitative.	[52,77,78,79]
**11**	**Protons**	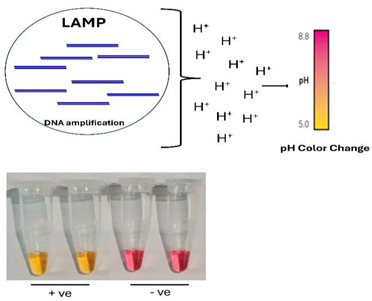	End point detection by the naked eye (pH indicator). Amenable to real-time detection with pH sensor. Single pot. Simple. Low cost.	Non-specific. Low sensitivity. Susceptible to spurious effects. Not amenable to multiplexing. Quantitative when pH meter is used.	[52,58,80]
**12**	**Bioluminescence**	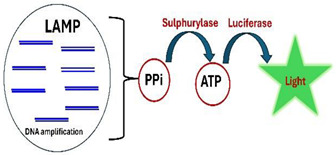	No need for excitation. Single pot. No background. Quantitative.	Substrate needed. No-specific. Not amenable to multiplexing. Continuous monitoring required. Cost.	[42,43,47]
**13**	**Lateral-Flow-Assay**	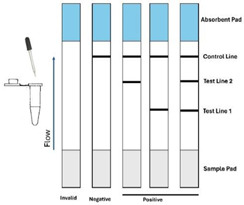	Simple. Low cost. Amenable to multiplexing.	Low sensitivity. Contamination risk. Time-consuming.	[52]
**14**	**Gel Electrophoresis/CE**	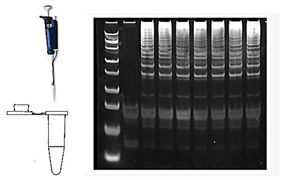		Non-specific. Low sensitivity. Not amenable to multiplexing. Contamination risk. Time-consuming. Poor quantification. Need for high voltage source and reader.	[52]

**Table 2:** Comparison of LAMP and Penn-RAMP techniques.

Features	LAMP	Penn-RAMP (RPA + LAMP)
**Temperature**	~63 °C	37 °C + 63 °C
**Sensitivity**	High (10^2^–10 genomic copies/reaction)	Very High (1 Copy/reaction) 10-fold better
**Specificity**	High	High
**Multiplexing**	≤3	~16

**Table 3:** LAMP and Penn-RAMP assays for respiratory tract viruses in chickens and the detection limit for each assay.

Viruses	LAMP Limit of Detection	Penn-RAMP Limit of Detection
**Infectious laryngotracheitis virus (ILTV)**	50 copies/µL [87] 353 copies/µL [88] 416 genomic copies/µL [36]	41 genomic copies/µL [36]
**Infectious Bronchitis Virus (IBV)**	1 EID_50_/mL [21] 666 genomic copies/µL [36] 2 × 10^2^ EID_50_/mL [20,98]	66 genomic copies/µL [36]
**Avian influenza virus (AIV)**	0.1 PFU/µL (H5) [106] 0.01 PFU/µL (H7) [109] 10 copies/µL (H9) [18]	NA
**Newcastle Disease virus (NDV)**	10^3^ EID_50_/mL [117] 5 genome copies/µL [99] 4.5 × 10^4^ copies/µL [17]	NA
**Avian metapneumovirus (aMPV)**	10 copies/µL [129]	NA

N/A = Not applicable.

## Data Availability

Not applicable.
